# Neurodegenerative Disorders and the Gut-Microbiome-Brain Axis: A Literature Review

**DOI:** 10.7759/cureus.72427

**Published:** 2024-10-26

**Authors:** Bindu Jyothi Dandamudi, Kathrina Antheia M Dimaano, Nensi Shah, Osamah AlQassab, Zainab Al-Sulaitti, Bhavana Nelakuditi, Lubna Mohammed

**Affiliations:** 1 Internal Medicine, California Institute of Behavioral Neurosciences & Psychology, Fairfield, USA; 2 Obstetrics and Gynecology, California Institute of Behavioral Neurosciences & Psychology, Fairfield, USA

**Keywords:** dysbiosis, gut, microbiome, neurodegenerative diseases, probiotics

## Abstract

Neurodegenerative diseases are severe, age-related conditions with complex etiologies that result in significant morbidity and mortality. The gut microbiome, a dynamic symbiotic environment comprising commensal organisms, represents the largest reservoir of these organisms within the human body. It produces short-chain fatty acids, endogenous signals, and neuroactive compounds, which can modulate neuronal function, plasticity, and behavior. Emerging evidence suggests that the gut microbiome plays a pivotal role in neurodevelopment, aging, and brain diseases, including Alzheimer’s disease, Parkinson’s disease, and stroke. Communication between the gut and brain occurs through a bidirectional channel known as the gut-microbiome-brain axis, which is being explored for therapeutic potential in neurodegenerative disorders. This literature review was conducted through a comprehensive search of five electronic databases - PubMed, Scopus, Ovid Medline, Cochrane Review, and Google Scholar - from inception to June 2024, focusing on English-language studies. Keywords included “gut-brain axis”, “microbiome dysbiosis”, “neurodegeneration”, and disorder-specific terms such as “Alzheimer’s disease” and “Parkinson’s disease”, paired with “gut microbiome”. The review examines current knowledge on the relationship between gut microbiota and neurodegenerative disorders, emphasizing potential mechanisms and therapeutic options. Results indicate that gut dysbiosis, characterized by microbial imbalance, is intricately associated with neurodegenerative disease pathogenesis by influencing immune responses, increasing blood-brain barrier permeability, and generating neurotoxic metabolites. Therapeutic approaches targeting the gut microbiome, including probiotics, prebiotics, and fecal microbiota transplantation, show promise in restoring microbial balance and slowing disease progression. However, further research is essential to validate these findings and develop effective clinical interventions.

## Introduction and background

Neurodegenerative diseases, such as Alzheimer’s disease, Parkinson’s disease, and amyotrophic lateral sclerosis, represent a significant public health concern due to their progressive nature and lack of effective treatments. These disorders are characterized by the gradual loss of neuronal structure and function, leading to severe cognitive, motor, and sensory deficits over time [1,2]. Despite extensive research, the exact pathophysiological mechanisms underlying these diseases remain elusive, largely due to their complex and multifactorial nature [3,4].

Recent advances in microbiome research have shed light on the potential influence of the gut microbiome on neurodegenerative disorders. The human gut microbiome, a diverse community of bacteria, viruses, fungi, and protozoa, plays a critical role in maintaining the host’s health by modulating various physiological processes, including immune response, metabolism, and neural function [5,6]. The concept of the gut-microbiome-brain axis, a bidirectional communication network between the gut and the central nervous system, has emerged as a pivotal area of study, linking gut health to brain function [7,8].

Evidence suggests that disruptions in the gut microbiome, termed dysbiosis, may contribute to the development and progression of neurodegenerative diseases by altering immune responses, increasing intestinal and blood-brain barrier permeability, and producing neurotoxic metabolites [9-11]. Studies have shown that gut dysbiosis can lead to increased systemic inflammation and neuroinflammation, both of which are key contributors to neurodegenerative disease pathology [12,13].

Furthermore, the gut microbiome is involved in the production of various neuroactive compounds, including neurotransmitters and short-chain fatty acids, which can directly influence brain function [14,15]. Understanding these interactions is crucial for developing novel therapeutic strategies that target the gut microbiome to prevent or mitigate neurodegenerative diseases.

This literature review aims to explore the current understanding of the relationship between gut microbiota and neurodegenerative disorders. Specifically, we will investigate the underlying mechanisms through which gut dysbiosis influences neurodegeneration and discuss potential therapeutic interventions aimed at restoring gut microbiome balance to improve neurodegenerative outcomes. Five electronic databases were searched (PubMed, Scopus, Ovid Medline, Cochrane Review, and Google Scholar) from inception to June 2024. No publication year restrictions were applied, and the review is restricted to literature published only in English. The keywords used in the literature search include “gut-brain axis”, “microbiome dysbiosis”, “neurodegeneration”, and disorder-specific terms like “Alzheimer's disease”, “Parkinson’s disease” paired with “gut microbiome”.

## Review

Gut-microbiome-brain axis

The gut-microbiome-brain axis is composed of the brain, endocrine system, immune system, autonomic outflow, and the gut microbiome [7]. The brain modulates gut function via the hypothalamic-pituitary-adrenal axis and the autonomic nervous system [16]. The gut influences the central nervous system through a multitude of microbiome-derived metabolites, neuroactive substances, and gut hormones. These regulators, through the enteric nervous system, vagus nerve, and immune system, exert their effects on the nervous system. Collectively, these pathways constitute the gut-microbiome-brain axis [7].

Gut Microbiome

The complex, nonpathogenic, active, diverse community of bacteria, fungi, archaea, parasites, and viruses that establish symbiotic relationships with the host constitutes the gut microbiome [4]. There is anatomical variation in the distribution of the gut microbiome throughout the digestive tract, with the stomach and small intestine containing a limited number of bacterial species, while the colon harbors a highly populated microbial community. Data from the MetaHit (European Union project on metagenomics of the human intestinal tract) and the Human Microbiome Project (a United States National Institutes of Health initiative) indicate that Proteobacteria, Firmicutes, Actinobacteria, and Bacteroidetes are the most dominant bacterial phyla [17,18].

Signaling Mechanisms Behind Gut-Microbiome-Brain Axis Communication

The gut-microbiome-brain axis is an advanced complex network connecting gut bacteria and the brain. It is crucial for maintaining a balance between the gastrointestinal, central nervous, and microbial systems. The communication between the gut-microbiome-brain occurs through both direct and indirect pathways involving chemical signals, neuronal routes, and the immune system [19].

Chemical Signaling

The chemical communication in the gut-microbiome-brain axis is an intricate process that involves both direct and indirect signals. Short-chain fatty acids and endogenous tryptophan are the two most extensively studied metabolites [19]. Short-chain fatty acids, produced from fermenting dietary fiber, impact the brain by affecting gene expression and the immune system. The short-chain fatty acids mainly include acetate (40-60%) and propionate (20-25%), which are predominantly produced by the Bacteroidetes phylum, and butyrate (15-20%), which is substantially produced by the Firmicutes phylum [20].

Short-chain fatty acids containing fewer than six carbon atoms play a crucial role in maintaining mucosal integrity and offer protection against gut inflammation and carcinogenesis. They affect the proliferation and mitosis of neural progenitor cells and play a vital role in brain development. Short-chain fatty acids are crucial for controlling a variety of central nervous system functions, including gene expression, neurotransmitter production and release, cell-cell contact, activation of microglia, and mitochondrial activity [6]. They are also implicated in glucose homeostasis, mucosal serotonin release, and lymphocyte function and influence learning and memory acquisition [6].

The gut microbiome also plays a significant role in the key metabolic pathways of essential amino acid tryptophan. The metabolic active products of tryptophan metabolism, such as indole-3-propionic acid, are vital for various physiological functions such as appetite regulation, inflammation, and gut immunity [6]. The gut microbiome is also crucial for the synthesis of serotonin, a neurotransmitter involved in regulating gut motility and systemic functions. These neuroactive metabolites communicate with the brain and the enteric nervous system by way of the intestinal mucosa and the vagus nerves [6]. The gut microbiome can also modulate the synthesis of other neurotransmitters such as dopamine, noradrenaline, gamma-aminobutyric acid, and acetylcholine, which impact the enteric nervous system, control gut motility, and influence the central nervous system [5].

Neuronal Pathways for Gut-Brain Interactions

Neuronal pathways create a direct physical link between the gastrointestinal tract and the central nervous system. The vagus nerve is the fastest and most direct way in this connection. The vagus nerve innervates muscle and mucosa layers of the gastrointestinal tract, detecting sensory signals and relaying them to the nucleus tractus solitarius. Nucleus tractus solitarius relays information it receives to the parabrachial nucleus, locus coeruleus, hypothalamus, amygdala, stria terminalis, ventral tegmental area, and medial septum regions in the brain. These areas control emotions and motivation. This pathway is vital for microbiota-brain communication. For example, administering *Lactobacillus rhamnosus *alters the expression of gamma-aminobutyric acid receptors in brain areas associated with emotions and fear, such as the amygdala and hippocampus. These changes are linked to anxiety-like behaviors in mice [21].

Immune System Pathways for Gut-Brain Interactions

There is a dense concentration of immune cells in the gastrointestinal tract, and they serve as an auxiliary defense against pathogens. The gut microbiota influences the production of both pro- and anti-inflammatory cytokines, which can then communicate with the brain through the circulatory system. Cytokines and chemokines can either be generated by immune cells within the brain or enter the central nervous system by crossing the blood-brain barrier. The gut microbiome is crucial in the development and function of the peripheral immune system [14].

Gut Microbiome and the Hypothalamic-Pituitary-Adrenal Axis

The hypothalamic-pituitary-adrenal pathway is an important non-neuronal route of communication within the microbiota-gut-brain axis. The gut microbiome activates the hypothalamic-pituitary-adrenal axis via cytokines like interleukin-1β, interleukin-6, tumor necrosis factor alpha, and various biologically active molecules [22]. Activation of the hypothalamic-pituitary-adrenal axis leads to the production of glucocorticoids, mediated through adrenocorticotropic hormone and corticotropin-releasing hormone. Cortisol enhances gluconeogenesis, protein, and fat metabolism and suppresses immune responses. This process regulates the activation of microglia and influences the brain’s inflammatory state [23].

Enteroendocrine Signaling

Widely distributed throughout the gastrointestinal tract, enteroendocrine cells have bidirectional regulatory effects on the brain. Enteroendocrine cells secrete gut hormones like ghrelin, glucagon-like peptide 1, glucose-dependent insulinotropic polypeptide, peptide YY, and cholecystokinin, which are implicated in gut-brain crosstalk [24].

Altered gut microbial profile and neurodegenerative disorders

Gut dysbiosis involves depletion in microbial diversity and beneficial bacteria like *Bacteroides* and Firmicutes, along with a proliferation in pathogenic bacteria such as Prevotellaceae and Enterobacteriaceae. This derangement can adversely affect the host’s health, leading to metabolic disorders, increased internal toxicity, systemic inflammation, and diminished levels of vital metabolites [25]. A diet high in refined foods, excessive alcohol consumption, additives, pollutants, stress, gastrointestinal surgeries, and prolonged use of antibiotics and other medications are various recognized factors contributing to gut dysbiosis [25]. Several studies established the link between the gut-microbiome brain axis alterations and the pathogenesis and progression of neurodegenerative disorders [10,26]. Gut dysbiosis leads to increased permeability across blood-brain and intestinal barriers, causing variations in the transport of gut microbes and their metabolites, which induces an inflammatory environment, disrupting the host immune regulation and modifying brain structure [10].

Role of Microbiome and Alzheimer’s Disease

Alzheimer’s disease is characterized by the accumulation of Aβ proteins as senile plaques in the brain’s extracellular spaces and hyperphosphorylated β-tau proteins forming neurofibrillary tangles inside neurons, leading to cognitive decline, memory loss, difficulties in verbal and motor activities, and dementia. Recent research suggests that certain bacteria from genera such as *Streptococcus*, *Staphylococcus*, *Salmonella*, *Mycobacteria*, *Klebsiella*, *Citrobacter*, and *Bacillus*, which are part of the gut microbiota, can produce Aβ proteins. These proteins can then misfold and accumulate into Aβ structures in the central nervous system, promoting neuroinflammation [12].

A recent study analyzing the gut microbiome of 25 Alzheimer’s disease patients demonstrated a decrease in microbial diversity. There was a notable reduction in Firmicutes and Actinobacteria, along with an increase in Bacteroidetes [27]. In patients with Alzheimer’s disease, the most prevalent families included Lachnospiraceae, Ruminococcaceae, and Bacteroidaceae. These microbial shifts correlate strongly with the pathological changes [27]. Cattaneo et al. observed a decline in the levels of anti-inflammatory bacteria such as *Eubacterium rectale *and an intensification in the levels of inflammatory species like *Escherichia *in elderly patients with Alzheimer’s disease [28].

Role of Microbiome and Parkinson’s Disease

Accumulation of Lewy bodies, primarily composed of alpha-synuclein and ubiquitin, in midbrain dopaminergic neurons is the pathognomic feature of Parkinson’s disease. Animal models indicate that abnormal α-synuclein deposition begins in the olfactory bulbs or enteric nervous system due to gut dysbiosis. This accumulation through synaptic transmission progresses to the dorsal motor nucleus of the vagus nerve, the substantia nigra pars compacta in the lower brainstem, and other central nervous system sites [11]. In Parkinson’s disease patients, there is an increased colonic expression of Toll-like receptor 4, CD3+ T cells, and pro-inflammatory cytokines, alongside a reduced abundance of short-chain fatty acid-producing bacteria [29].

Role of Microbiome and Multiple Sclerosis

Multiple sclerosis is a neurodegenerative disorder caused by gradual demyelination and axonal damage leading to neurodegeneration via autoimmune mechanisms. Clinically, it manifests by sensory, visual, autonomic, and cognitive impairments, along with ataxia, muscle spasms, paralysis, bladder and bowel dysfunction, and fatigue [30]. Recent evidence suggests that gut dysbiosis is a key factor in multiple sclerosis neuropathogenesis [31,32]. Specifically, the relapsing/remitting form of multiple sclerosis is associated with a decreased abundance of anti-inflammatory bacteria and an increased abundance of pro-inflammatory bacteria, which regulate immune cells including regulatory T cells, interleukin 10-secreting CD4+ T cells, regulatory B cells, tolerogenic dendritic cells, and suppressive macrophages [13]. Clinical studies report that the gut microbial profile of multiple sclerosis patients differs from that of healthy individuals [33]. Patients with relapsing-remitting multiple sclerosis show decreased levels of *Parabacteriodes distasonis* and *Faecalibacterium *compared to healthy controls [32].

Role of Microbiome and Amyotrophic Lateral Sclerosis (Lou Gehrig’s Disease)

Amyotrophic lateral sclerosis, formerly known as Lou Gehrig’s disease, is a neurological disorder marked by the gradual loss of motor neurons in the brain and spinal cord, resulting in muscle weakness and paralysis [2]. Misfolded proteins, particularly ubiquitinated cytoplasmic inclusions, are found in neurons and glia in amyotrophic lateral sclerosis. Glutamate excitotoxicity, mitochondrial dysfunction, redox imbalance, RNA metabolism changes, and dysregulated autophagy are all implicated in the pathogenesis [15].

Recent studies highlight a strong link between gut dysbiosis and amyotrophic lateral sclerosis [34,35]. Specifically, amyotrophic lateral sclerosis pathogenesis is associated with alterations in gut microbiota composition, impaired metabolism, an altered immune response, and the production of neurotoxins by Clostridia species that can induce brain damage [34]. A metagenomics study found that patients with amyotrophic lateral sclerosis exhibit a reduced Firmicutes/*Bacteroides *(F/B) ratio, indicating gut dysbiosis [36]. Additionally, there is a significantly increased abundance of the genus Dorea and a decreased abundance of the genera *Oscillibacter*, *Anaerostipes*, and Lachnospiraceae compared to healthy controls [36].

Role of Microbiome and Huntington’s Disease

Huntington’s disease is a neurodegenerative movement disorder characterized by involuntary movements of the head, neck, limbs, and face, caused by genetic anticipation of the cytosine-adenine-guanine repeat in the Huntington gene [37]. Clinical features include cognitive, psychiatric, and motor impairments, unintended weight loss, skeletal muscle atrophy, and gastrointestinal dysfunction. The early pathological feature is the degeneration of neurons in the putamen, caudate, and cerebral cortex. Altered gut metabolites have been observed in Huntington’s disease patients and transgenic animals, suggesting gut microbiota changes before disease onset. In Huntington’s disease mouse models, gastrointestinal dysfunction caused unintended weight loss. A recent study on the R6/1 transgenic mouse model of Huntington’s disease found altered gut microbial composition, with increased Bacteroidetes and decreased Firmicutes levels at 12 weeks of age. This gut dysbiosis is coordinated with impaired weight gain despite higher food intake and motor deficits [38].

Therapeutically targeting the microbiome-gut-brain axis

There is increasing evidence that links gut microbiome to brain disorders and therapeutic usage of probiotics and synbiotics to address gut dysbiosis could be a promising approach for managing neurodegenerative disorders [8].

Probiotics (living microorganisms that, when ingested, provides a health benefit) enhance intestinal and immune homeostasis by restoring the gut microbiome [39], and demonstrate neuroprotective effects in central nervous system disorders by amplifying the production of gamma-aminobutyric acid and glutamate [40]. Probiotics with their anti-inflammatory and antioxidant properties help to reduce the incidence of neurodegenerative diseases. Probiotics such as *Escherichia coli*, *Lactiplantibacillus plantarum*, and *Bifidobacterium pseudocatenulatum *improve memory dysfunction and cognitive dysfunction [39]. Clinical trials utilizing probiotics are summarized in Table 1.

**Table 1:** Summary of the human clinical trials using probiotics

Author	Study objective	Disease	Type of study	Conclusion
Akbari et al. [41]	To assess if probiotic supplementation helps to improve cognitive and metabolic disorders	Alzheimer’s disease	Randomized, double-blind trial	Probiotics for 12 weeks positively improved cognitive function
Agahi et al. [42]	To assess the cognitive status of the people treated with a mixture of probiotic bacteria	Alzheimer’s disease	Randomized, double-blind, and placebo-controlled clinical trial	We concluded that the cognitive and biochemical functions are insensitive to the probiotic supplementation
Tamtaji et al. [43]	To determine the effects of probiotics and selenium co-supplementation on cognitive function	Alzheimer’s disease	Randomized, double-blind, controlled clinical trial	Probiotic and selenium co-supplementation improved cognitive function
Hwang et al. [44]	This study investigated the efficacy and safety of *Lactobacillus plantarum* C29-fermented soybean (DW2009) as a nutritional supplement for cognitive enhancement	Alzheimer’s disease	Multi-center, randomized, double-blind, placebo-controlled clinical trial	Improved cognitive function, and increased serum brain-derived neurotrophic factor level
Akbari et al. [45]	To assess if probiotics help to improve cognitive disorders in Alzheimer’s patients	Alzheimer’s disease	Double-blind, randomized controlled trial	The study demonstrated that probiotic consumption positively affects cognitive function
Tamtaji et al. [46]	To assess the impacts of probiotic supplementation on movement and metabolic parameters	Parkinson’s disease	Randomized, double-blind, placebo-controlled trial	Our study showed that probiotic consumption had useful impacts on the Movement Disorders Society-Unified Parkinson’s Disease Rating Scale
Asaoka et al. [47]	Investigated the effect of the probiotic strain *Bifidobacterium breve* MCC1274 (A1) on cognition and preventing brain atrophy	Alzheimer’s disease	24-week randomized, double-blind, placebo-controlled trial	Probiotic consumption suppressed brain atrophy progression and prevented cognitive impairment
Di Gioia et al. [48]	To study the impact of probiotic supplementation on the gut microbiome and disease progression	Amyotrophic lateral sclerosis	Double-blind, placebo-controlled randomized control trial	Probiotic supplementation did not influence disease progression

Fecal microbiota transplantation involves transferring fecal matter encompassing the gut microbiome from a source to a receiver with a disorder linked to gut dysbiosis using methods like enema, nasogastric tube, gastroenteric tube, or endoscopy. The goal is to restore a healthy diversity of the microbial community. Fecal microbiota transplantation can affect the production of gut microbial metabolites, such as serotonin, short-chain fatty acids, and gamma aminobutyric acid receptor agonists, whereas a reduction of pro-inflammatory gut bacteria may decrease neuroinflammation and cerebral oxidative stress. It has emerged as a promising approach to address gut dysbiosis associated with neurodegenerative disorders. Clinical trials utilizing fecal microbiota are summarized in Table 2.

**Table 2:** Summary of the human clinical trials using fecal microbiota

Author	Study objective	Disease	Type of Study	Conclusion
Chen et al. [49]	This study aimed to investigate the safety and efficacy of fecal microbiota transplantation for cognitive impairment treatment	Alzheimer’s disease	An open-label, single-arm trail	Fecal microbiota supplementation improved cognitive function in mild cognitive impairment
DuPont et al. [50]	It is a pilot clinical study looking at the safety of fecal microbiota transplantation and the improvement of symptoms in Parkinson’s disease	Parkinson’s disease	A randomized double-blind, placebo-controlled clinical pilot study	Fecal microbiota transplantation improved subjective motor and non-motor symptoms
Segal et al. [51]	We aimed to determine whether fecal microbiota transplant is efficacious in treating constipation, motor, and non-motor symptoms	Parkinson’s disease	Single arm, case series	Fecal microbiota transplantation improved motor and non-motor symptoms

## Conclusions

This literature review emphasizes the complex bidirectional relationship between the gut microbiome and the brain, where microbial populations influence neurological and psychiatric conditions through pathways like the vagus nerve, immune system modulation, and metabolite production. Disruptions in microbial populations have been linked to various neurodegenerative diseases. Therapeutic interventions, such as probiotics, prebiotics, and dietary modifications, are explored for their potential to restore healthy microbial balance and, in turn, improve mental and neurological health outcomes.

Ongoing research studies are steadily unveiling the elaborate relations between the gut microbiome and their symbiotic relationship with human hosts. Gaining a comprehensive understanding of how the microbiome influences neurodegenerative diseases like Alzheimer’s disease and Parkinson’s disease will significantly enhance efforts in their prevention, treatment, and management. As our knowledge of the gut microbiome strengthens, innovative strategies can be devised for noninvasive prognostic microbiome-based biomarkers for various neurodegenerative diseases. Future research directions may include randomized clinical trials to support the clinical application of prebiotics, probiotics, synbiotics, and fecal microbiota transplantation for managing neurodegenerative disorders.
